# Spatiotemporal analysis of agricultural drought variability in the uMkhanyakude District Municipality, KwaZulu-Natal

**DOI:** 10.1007/s10661-024-13527-9

**Published:** 2025-01-20

**Authors:** Jabulile Happyness Mzimela, Inocent Moyo, Thulani Tshabalala

**Affiliations:** 1 Department of Geography and Environmental Studies, University of Zululand, Empangeni, KwaZulu-Natal South Africa; 2 Department of Life and Consumer Sciences, University of South Africa, Johannesburg, South Africa

**Keywords:** Drought monitoring, El Niño, Climate change, Vegetation indices, South Africa

## Abstract

Exploring drought dynamics has become urgent due to unprecedented climate change. Projections indicate that drought events will become increasingly widespread globally, posing a significant threat to the sustainability of the agricultural sector. This growing challenge has resulted in heightened interest in understanding drought dynamics and their impacts on agriculture. uMkhanyakude District Municipality (UKDM) has experienced substantial drought occurrences, and 95% of rural dwellers within the district depend on small-scale agriculture, social security grants, and remittances for their livelihoods. Hence, there is a critical need for spatiotemporal assessments of drought within the district to fully comprehend the severity and spatial distribution of these events. This study addressed this need by assessing vegetation variability and agricultural drought occurrences across the UKDM from 2002 to 2023, by leveraging key vegetation health indices—namely, the Vegetation Condition Index, Temperature Condition Index, and Vegetation Health Index (VHI). The results identified major historical droughts, including episodes in 2002–2004 and 2015–2016 linked to El Niño events. Moreover, the findings revealed localised vulnerability to drought, although severe drought was limited at the district level. Moderate drought conditions characterised most months across Mtubatuba, Umhlabuyalingana, Jozini, and Big 5 Hlabisa local municipalities, ranging from 40.34% in Jozini to 59.75% in Umhlabuyalingana. No drought conditions occur less frequently across the district, ranging from 0.89% in Jozini to 7.33% in Mtubatuba, indicating limited periods of optimal vegetation health. This pattern suggests that certain areas within the district are particularly susceptible to drought, potentially threatening agricultural productivity and food security. The study highlights the efficacy of vegetation indices in capturing known drought events, underscoring their utility, especially in regions where ground-based data may be scarce. This spatiotemporal assessment provides an enhanced understanding of agricultural drought patterns to inform drought-related decision-making and adaptation in the agricultural sector. Given the consistent vulnerability identified, government support should be directed toward drought-prone areas, particularly through enhanced water resource management and infrastructure investment. Targeted measures are particularly recommended for areas with persistently low VHI values, such as the inland western regions of Jozini. Such efforts will strengthen resilience and sustainability in agricultural practices, safeguarding livelihoods and food security.

## Introduction

Understanding the impact of climate-related hazards on agricultural systems is gaining momentum due to the growing frequency of drought (Kiumbuku et al., 2020; Lottering et al., 2021). Approximately 60% of the global population is affected by drought, highlighting its status as a widespread and complex hazard (Kiumbuku et al., 2020). Drought impacts vary across different geographies and sectors and are influenced by socio-environmental factors (Liguori et al., 2021). Over the past 50 years, African countries have experienced increases in drought frequency, intensity, and spatial extent (Ncoyini-Manciya, 2021). In southern Africa, climate change has led to more frequent droughts, which are expected to worsen with rising temperatures and decreasing rainfall (Shayanmehr et al., 2020). This situation underscores the urgent need for adaptation strategies and enhanced resilience within the agricultural sector. The reliance on rain-fed systems in Africa’s and South Africa’s agricultural sectors in particular increases their vulnerability to drought (Abegunde et al., 2020). In South Africa, a great portion of the rural population relies on agriculture for sustenance, employment, and income, making the sector a pivotal component of the nation’s economy and a key contributor to foreign exchange (Hlatshwayo et al., 2021; Nkamisa et al., 2022). For instance, in the fiscal year 2021, South Africa’s agricultural sector contributed around 10% of the nation’s total export earnings, valued at $12.0 billion (International Trade Administration, 2024).

Several studies have characterised drought in South Africa and within KwaZulu-Natal (KZN) such as those by Lottering et al. (2022) and Ndlovu and Demlie (2020). For example, Ndlovu and Demlie’s (2020) study found that drought severity and frequency have increased across KZN, with the northern region—including uMkhanyakude District Municipality (UKDM)—being most affected during extreme drought conditions. Despite these insights, a gap remains in understanding how vegetation in this region responds to drought. Such insights are essential for supporting agricultural planning, improving drought risk management, and assessing food security. To address this gap, the present study analysed vegetation variability and agricultural drought occurrence across the UKDM from 2002 to 2023 using satellite remote sensing and Geographic Information Systems (GIS) technologies. This research has the potential to influence policy and adaptation strategies in the district.

In particular, the satellite-derived vegetation indices utilised in this paper include the Normalised Difference Vegetation Index (NDVI), Vegetation Condition Index (VCI), Temperature Condition Index (TCI), and Vegetation Health Index (VHI). NDVI measures overall vegetation greenness; VCI identifies deviations in vegetation health from normal conditions; TCI detects temperature-related vegetation stress; and VHI combines VCI and TCI as a composite indicator of overall vegetation health (Kogan, 2001). These indices enhance the study’s ability to monitor vegetation health, assess drought severity, and inform strategies for agricultural adaptation and resilience in the face of climate change and variability. Moreover, they serve as pivotal tools for detecting moisture stress and evaluating changes in vegetation health over time, enabling the identification of emergent agricultural drought conditions and facilitating the allocation of support.

This paper uses these indices to augment our understanding of drought evolution and impacts, laying a foundation for quantifying vulnerabilities and crafting tailored interventions across impacted areas. To assess the spatiotemporal variability of agricultural droughts in the UKDM, the article is structured as follows: The second section offers a literature review on drought, focusing on agricultural drought. The third section describes the geographical and socio-economic context of the study area. The fourth section details the methodologies employed in this paper. The fifth section presents the study’s findings and discusses their implications. Lastly, the sixth section synthesises the key findings and concludes the study.

## Global drought dynamics and regional impacts

Drought is partly created or shaped by changes in regional climate, local catchment characteristics, water consumption, institutional systems, and socio-technical dynamics (Taylor et al., 2009). Socio-technical dynamics encompass the interactions between governance structures, social norms, everyday practices, and technical water management which collectively shape the course, size, and distribution of drought events. While drought has inherent natural properties, researchers have developed operational classifications to address its complexity. These classifications consider the affected biophysical and socio-economic systems and include meteorological drought (groundwater deficit due to low precipitation), agricultural drought (insufficient soil moisture for crops), hydrological drought (reduced streamflow), and socio-economic drought (impact on society) (Cao et al., 2022; Tian et al., 2022). A single area can experience multiple drought types simultaneously, underscoring the multifaceted nature of this hazard (Lin et al., 2023). A region’s susceptibility to drought is determined by its physical characteristics, socio-economic conditions, and the efficacy of its adaptive strategies (Masroor et al., 2023). The severity of drought is contingent upon its intensity, duration, and the community’s resilience and preparedness (Chivangulula et al., 2023). Additionally, drought can amplify the risk of other hazards, such as heatwaves and floods (Ndlovu & Mamba, 2023). Recognising the complex interplay of these factors is essential for developing comprehensive risk assessments and integrated management strategies.

Drought has been recorded on every continent, with 364 events documented globally (Dikshit et al., 2022). An unprecedented concentration of these events occurred in 2015 (Dikshit et al., 2022). In Europe, significant periods of soil moisture drought since 1766 include the years 1857–1860, 1920–1922, 1947–1948, 1975–1977, 2003–2004, 2015–2016, and 2018–2020 (Rakovec et al., 2022). Germany, in particular, experienced significant droughts in 2003, 2015, 2018, and 2019 (Sodoge et al., 2023). Similarly, North America’s history of droughts spans millennia, some of which dwarf the 1930s, 1950s, and 1998–2014 drought episodes in intensity and duration (Heim, 2017). In India, major soil moisture drought periods from 1870 to 2016 include 1876–1882, 1895–1900, 1908–1924, 1937–1945, 1982–1990, 1997–2004, and 2011–2015 (Mishra et al., 2019). The Xinjiang region in China also experienced its most severe drought conditions in 1962, 1974, 1977, 1981, 2000, 2001, 2006, 2008, 2011, and 2015 (Yao et al., 2019). Across Africa, severe droughts occurred in 2003, 2005, 2008, 2013, 2015, and 2019, impacting over half of the continent’s regions (Khan & Gilani, 2021). Drought frequently devastates Southern Africa (Ayugi et al., 2022), with the potent El Niño event of 2015/2016 marked as one of the worst in its history (Maponya & Mpandeli, 2017). This event led to the most severe drought in the 116-year record (1900 to 2016) (Chivangulula et al., 2023), during which the region experienced all four types of droughts concurrently (Orievulu et al., 2022).

South Africa is recognised as a water-stressed country (Ndlovu & Demlie, 2020), consistently confronting challenges posed by drought conditions (Meza et al., 2021). Historical records highlight major drought events in 1973–1974, 1982–1984, 1991–1992, 1994–1995, 2004–2005, 2008–2009, 2015–2016, and, most recently, 2018–2020 (Meza et al., 2021; Ruwanza et al., 2022). In 2015, the country experienced its driest year since 1904 (Slayi et al., 2023). Research by Ndlovu and Demlie (2020) identified diverse drought conditions in KZN, with periods of slight to moderate dryness in 1970–1973, 1997, 1981, 1983, 1986, 1990–1991, 1993–1994, 2001, 2004, 2009–2011, and 2016. Moreover, very dry to extremely dry conditions were prevalent in 1979–1980, 1992, 2002–2003, 2014, and 2015 (Ndlovu & Demlie, 2020). Orievulu et al. (2022) confirmed these findings, listing drought occurrences in KZN for 1900, 1902–1904, 1912, 1928, 1931, 1941, 1945, 1992, 2003, 2014, and 2015. An analysis of drought conditions within the UKDM revealed varying degrees of drought severity. Specifically, 1997 and 1998 experienced moderate to severe droughts; 2002 was marked by extreme drought conditions, and 2015 saw moderate and extreme droughts (Gwala, 2018). Moreover, drought-induced states of disaster were declared in KZN in 2014 and 2016 (Provincial Gazette No. 1288 dated 17 December 2014 and Provincial Gazette No.1600 dated 08 February 2016). Moreover, in 2018, the drought was severe and prompted a national disaster declaration (Government Notice 107/2017, published in Government Gazette 41,439 on 13 February 2018).

## Agricultural drought impacts

Agriculture’s susceptibility to climate variability positions it as the primary economic sector that bears the brunt of drought conditions, often experiencing the most immediate and severe impacts (Ayugi et al., 2022). Agricultural drought is recognised globally as one of the costliest hazards, causing widespread damage and affecting more people than any other hazard (Bahta & Myeki, 2022). The agricultural sector consumes large quantities of water (Ward et al., 2020), a necessity primarily driven by the essential role of soil moisture in plant growth, making it especially susceptible to drought (Lee et al., 2022). Accordingly, drought impacts are more pronounced in this sector (Holman et al., 2021; Zhang et al., 2022). Meteorological drought, high temperatures, and strong winds, which lead to high evapotranspiration, drive agricultural drought by reducing soil moisture to levels inadequate for plant growth (Sharma et al., 2022). Researchers note that agricultural drought is more complex and less understood than other drought types due to intricate vegetation and climatic interactions (Cao et al., 2022; Sandeep et al., 2021). This complexity, combined with its impact on food systems, underscores the focus on agricultural drought in this paper, as it poses a significant threat to global food security (Kiumbuku et al., 2020; Lottering et al., 2021; Orievulu et al., 2022). Additionally, the frequent and lasting effects of agricultural drought lead to environmental and socio-economic challenges (Cao et al., 2022).

The impacts of agricultural drought are extensive, often resulting in reduced or failed crop yields, increased pest infestations, and plant diseases (Nkamisa et al., 2022). Regionally, agricultural drought exacerbates inequality, raises food prices, heightens food insecurity, increases unemployment, and drives migration (Nemeth, 2020). At the national level, drought can reduce tax revenue and weaken the economy (Nemeth, 2020). Globally, a single drought event can lower the agricultural gross domestic product by 0.8%, with approximately 80% of the economic losses in developing countries attributed to agricultural drought (Bahta & Myeki, 2022). In Germany, for example, drought caused a 17% decrease in agricultural yields in 2018, alongside other adverse effects on ecosystem services (Sodoge et al., 2023). In Northern China, recurring droughts have severely impacted the agricultural sector, causing population migration (Alkhalidi et al., 2023). The 2008–2009 drought near the eastern Tianshan Mountains and southern Xinjiang led to the loss of 1.22 million hectares of crops and 28 million hectares of grasslands, with direct economic losses reaching one billion yuan (Yao et al., 2019).

The situation is equally grim in Africa, where drought negatively impacts crop and livestock farming (Slayi et al., 2023). As a case in point, South Africa’s crop production was cut by 40% during the 1991–1992 drought period (Baudoin et al., 2017). Moreover, during the 2015 extreme drought in South Africa, about 2.7 million households experienced water shortages, and agricultural production declined (Lottering et al., 2021), resulting in massive economic losses worth US$250 million (Vetter et al., 2020; Xulu et al., 2019). The 2015–2017 drought in South Africa further reduced maize production by over 30% and prompted severe water restrictions in major cities like Cape Town (Baudoin et al., 2017). Within the specific context of KZN, the 2014–2016 drought episode caused economic losses exceeding ZAR 10 billion in livestock production alone, a setback likely correlated with severe food insecurity in the province (Ruwanza et al., 2022). Cattle farmers in KZN lost 43% of their herds and 29% of their goats during this drought (Bahta & Myeki, 2022). These losses have motivated increased research output (Phaduli, 2018), raised public awareness about the severe effects of inadequate and poorly maintained water infrastructure (Jordaan, 2017), and prompted adaptation-related responses (Adisa et al., 2019).

## Description of the study area

**Fig. 1:**
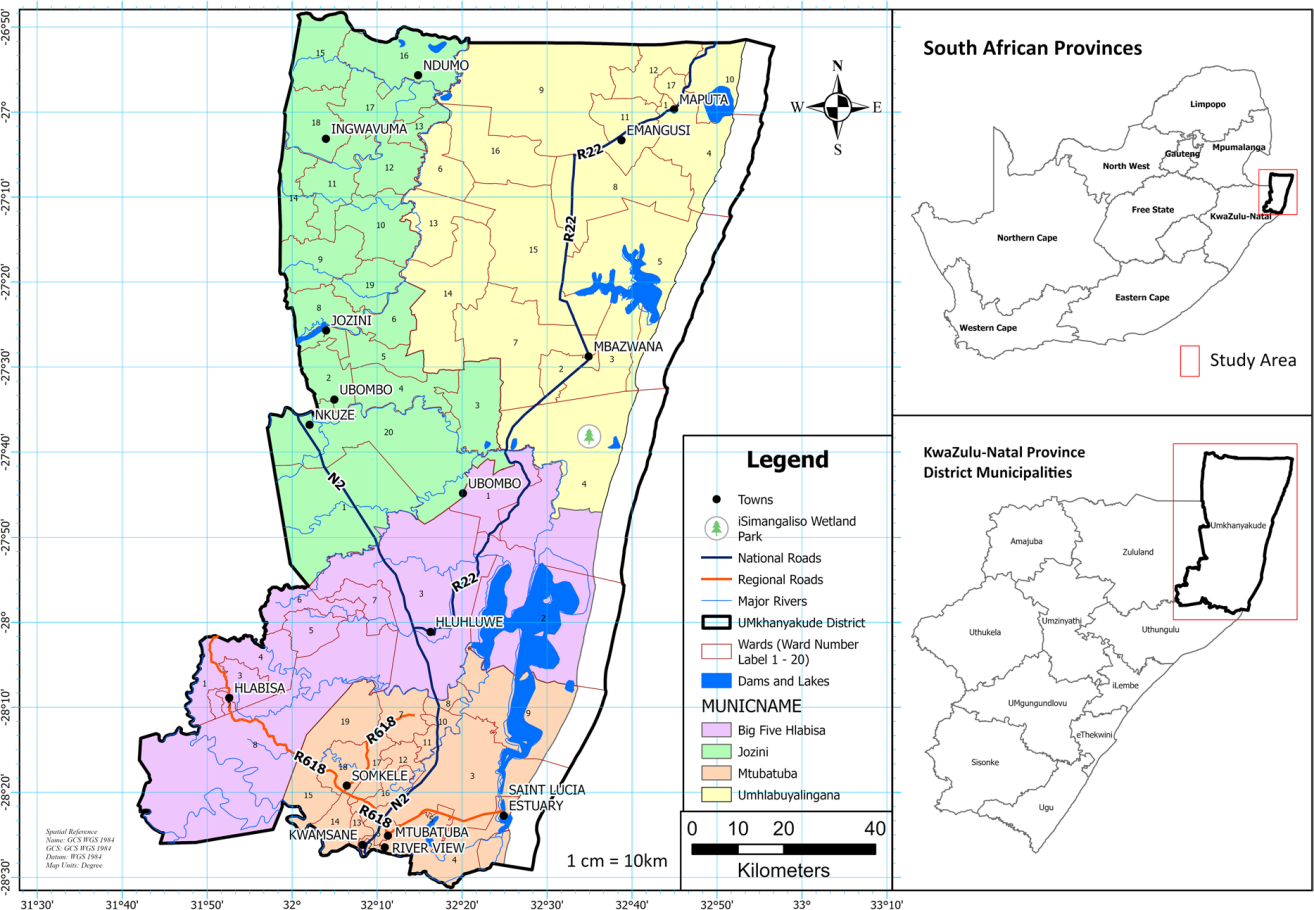
uMkhanyakude District Municipality within South Africa

The study area, UKDM (Fig. 1), is a predominantly rural district (32,014489; −27,622,242) (Sichewo et al., 2020) and ranks as the second-largest district in KZN, covering 13,855 km^2^ (Dlamini et al., 2017). The district is characterised by a temperate climate, with warm to hot summers and mild winters—a climatic pattern influenced by the southward flow of the warm Agulhas Current (Ezemvelo KZN Wildlife, 2014). Average daily minimum temperatures exceed 10 °C, while average daily maximum temperatures surpass 20 °C (Morgenthal et al., 2006). Precipitation in the district is predominantly seasonal, occurring during the summer months between October and March (Dlamini et al., 2021; Morgenthal et al., 2006). The annual rainfall exhibits variability across the district, with averages ranging from 671 to 1002 mm (Morgenthal et al., 2006), reflecting the diverse climatic conditions influencing the region’s environmental and agricultural dynamics.

The district falls within the Mfolozi/Pongola Primary Catchment Area, a hydrological catchment that extends across borders to include regions within Eswatini and Mozambique (DALRRD, 2016). It features a varied landscape, encompassing mountains, ridges, low-lying plains, and an array of freshwater streams and coastal waterways (Orievulu & Iwuji, 2021). The district’s vegetation type is classified as the Maputaland coastal thicket (Akweni et al., 2020), and land use is mainly rural, comprising expansive natural landscapes (DALRRD, 2016), agricultural land, along with a small proportion of high-density settlements (Ezemvelo KZN Wildlife, 2014).

Agriculture plays a crucial role in shaping the socio-economic and cultural landscape of the UKDM community (Orievulu et al., 2022). In the region, small-scale agriculture primarily involves sugarcane, cotton, vegetable farming, and livestock rearing (DALRRD, 2016), with these activities mainly concentrated in the Pongola floodplain and the coastal lake wetland systems (EDTEA, 2021). Conversely, commercial agriculture, including sugarcane, pineapple, forestry, livestock, game, citrus, and vegetable farming, is concentrated along the narrow farmland corridor adjacent to the N2 highway (Bigen Africa, 2016). Additionally, various vegetables and field crops, such as potatoes, sweet potatoes, chillies, tomatoes, cabbage, dry beans, and maize, are cultivated across the district (DALRRD, 2016).

The UKDM is one of the regions in South Africa most severely affected by climate change-induced water scarcity (Patrick, 2021). The district’s susceptibility is attributed to several factors, including high poverty levels (UMkhanyakude District, 2014), high dependence on rain-fed small-scale farming, and existing water scarcity issues (Mulopo & Chimbari, 2021). According to Mzimela and Moyo (2024), droughts occur frequently in the district and have become deeply embedded in the collective memory of farmers, adversely affecting agricultural production. Moreover, only 26% of the households within the district report always having sufficient food stocks, while food insecurity has intensified for the rest of the population (Chikafu & Chimbari, 2020). The historical trend of increasing drought occurrences, along with projections of heightened severity and frequency of such events, justifies the necessity for drought assessments (Ndlovu & Demlie, 2020). These assessments are crucial for developing mitigation and adaptation strategies to safeguard agricultural productivity and, by extension, the UKDM community’s livelihoods against climate change and variability.

## Methodological framework

Remote sensing is a powerful tool for identifying and monitoring drought conditions (Wardlow et al., 2012). One of its primary advantages is the ability to provide consistent data coverage in areas lacking in situ measurements or where ground data is difficult to obtain (Mukhawana et al., 2023). Furthermore, AghaKouchak et al. (2015) note that satellite-based measurements capture key drought-related variables, such as precipitation, soil moisture, and vegetation health, providing extensive spatial coverage and frequent temporal observations that complement ground-based data. Additionally, the frequent revisit times of satellite sensors enable timelier drought detection. Thus, remote sensing and GIS strengthen drought monitoring and impact evaluation, both essential for improved drought preparedness and response (Tadesse et al., 2017). Hence, this study applies remote sensing and GIS techniques to monitor agricultural drought patterns across the UKDM, utilising satellite-derived vegetation indices such as NDVI, VCI, TCI, and VHI.

**Fig. 2:**
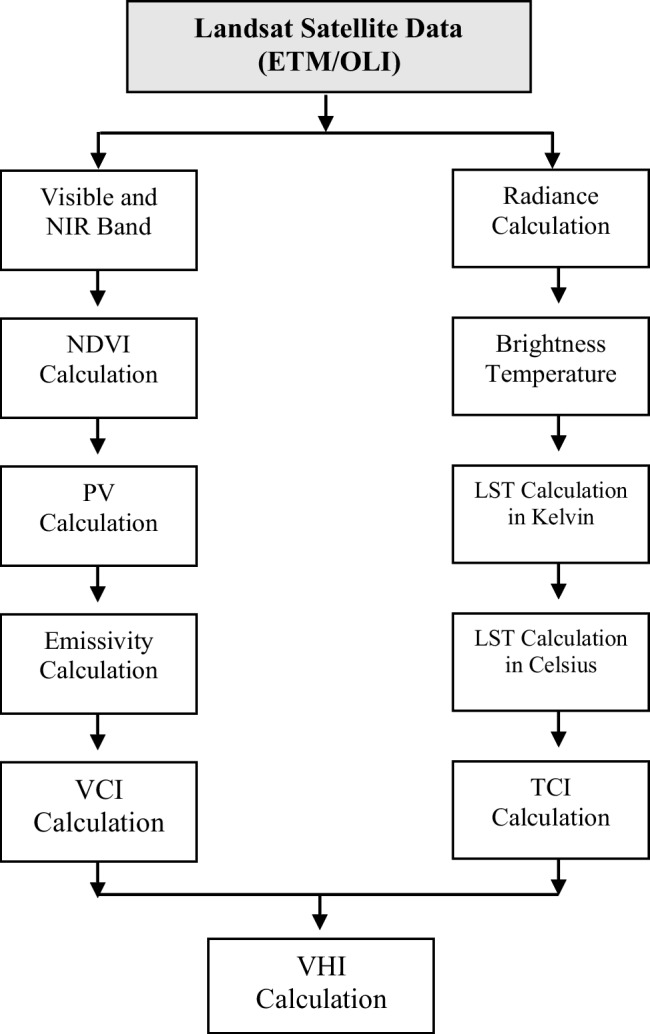
Methodological framework adopted

The NDVI was the pioneering remote-sensing vegetation index developed to monitor agricultural drought by detecting changes in vegetation greenness and density (Chang et al., 2021). The VCI, derived from the NDVI (Chere et al., 2022), is a useful tool for assessing agricultural drought onset, duration, cessation, intensity, and effects on vegetation (Mukhawana et al., 2023). Similarly, the TCI measures temperature-induced vegetative stress by assessing changes in Land Surface Temperature (LST) over a specified period (Chere et al., 2022). The VHI is computed as a weighted average of the VCI and TCI, offering a more comprehensive assessment of drought impact on vegetation (Lee et al., 2021; Sandeep et al., 2021). This study prioritises the VHI as the main index, due to its ability to capture vegetation responses that reflect local biophysics and climatic factors (Zeng et al., 2022). By integrating the VCI and TCI, VHI offers a nuanced perspective that accounts for the combined influence of moisture and thermal stress on vegetation, making it an essential tool for drought monitoring across regions with diverse climatic profiles. The VHI has been utilised across a range of applications at global, regional, and national scales, including identifying drought events, assessing drought severity and duration, issuing early warnings of drought conditions, and monitoring crop yield and production during the growing season (Karnieli et al., 2006). The adopted methodology (Fig. 2) consists of three main phases: (i) acquisition of satellite data for the UKDM from remote sensing platforms; (ii) analysis of this satellite data to derive vegetation health indices, specifically the VCI, TCI, and VHI; and (iii) spatial and temporal analysis of these indices using Google Earth Engine (GEE) to study trends and patterns of drought progression across the UKDM over the study period.

First, the NDVI was calculated from Landsat reflectance values in the red and near-infrared bands using the following equation adopted from Rouse et al. (1974):

1 $$\mathrm{NDVI}=(\mathrm{NIR}-\mathrm{Red})/(\mathrm{NIR}+\mathrm{Red})$$

where NIR and Red refer to spectral reflectance in the near-infrared and red regions, respectively.

NDVI values range from − 1 to 1, with higher values indicating greater vegetation greenness and health.

The VCI is then computed from the NDVI values as follows (Kogan, 2001):

2 $$\text{VCI}=\left(\text{NDVI}-{\text{NDVI}}_\text{min}/{\text{NDVI}}_\text{max}-{\text{NDVI}}_\text{min}\right)\times100$$

where NDVImin and NDVImax represent the historical minimum and maximum NDVI values for a given pixel and period. VCI ranges from 0 to 100, with higher values denoting better vegetation conditions with less drought stress.

Similarly, the TCI is derived from LST satellite observations (Wan et al., 2004):

3 $$\text{TCI}\:=\:({\text{LST}}_{\text{max}}-\text{LST}/{\text{LST}}_{\text{max}}-{\text{LST}}_{\text{max}})\:\times\:100$$

where LSTmin and LSTmax are historical min/ max LST. TCI also ranges from 0 to 100, with lower values signalling higher temperature stress on vegetation.

Finally, VHI integrates VCI and TCI using a weighted average (Kogan, 2001):

4 $$\text{VHI}\:=\:\mathrm a\:\times\:\mathrm{VCI}\:+\:(1\:-\:\mathrm a)\:\times\:\mathrm{TCI}$$

where a is a coefficient controlling the weights and *a* = 0.5.VHI combines vegetation greenness and thermal stress information to indicate overall vegetation health from 0 to 100. Lower VHI points to greater agricultural drought severity.

Additionally, the VCI and TCI values were normalized to ensure their contributions to the VHI were balanced without the need for explicit parameter adjustments. This approach ensures that the VHI accurately reflects the combined impact of vegetation and temperature conditions in a fair and standardized way. The calculated VHI values were classified into different drought severity categories following Cai et al. (2018), as shown in Table 1.

**Table 1:** Drought classification using VHI

VHI range	Drought category	Description
40 < VHI	No drought	Healthy vegetation with no moisture stress
30 < VHI ≤ 40	Mild drought	Slight vegetation stress, soil moisture deficit
20 < VHI ≤ 30	Moderate drought	Moderate vegetation stress reduced productivity
10 < VHI ≤ 20	Severe drought	High vegetation stress, moisture deficiency, crop damage likely
10 ≤ VHI	Extreme drought	Extreme vegetation moisture stress, plant mortality possible, crop failure

Source: adapted from Cai et al., 2018

The analysis and processing of satellite data to derive vegetation health indices were conducted using GEE and Python programming languages. This approach enabled a streamlined, automated workflow that supports continuous, reproducible monitoring of vegetation health and therefore provides insights into long-term vegetation trends and drought impacts across the UKDM. GEE provided access to the MODIS sensor images, which are hosted on the Terra and Aqua satellite platforms (launched in December 1999 and May 2002, respectively). This infrastructure facilitated the study’s focus on data spanning from 2002 to 2023. GEE’s platform allowed efficient handling and processing of extensive image collections, accommodating the large spatial and temporal scale of the study (Gorelick et al., 2017). Python’s robust packages—NumPy and Pandas—were employed for data manipulation and time-series analysis of vegetation health indices (Harris et al., 2020; McKinney, 2010). Python scripting allowed the automation of GEE workflow steps like masking clouds and calculating indices for each scene. This combination of GEE and Python provided a scalable, flexible framework for generating vegetation indices from raw satellite imagery.

Vegetation indices serve as valuable indicators of vegetation health and vigour but have limitations. For example, distinguishing drought-induced vegetation stress from changes caused by other local factors such as floods, fires, pest outbreaks, or land use change is challenging, as these disturbances also affect crop health but are not accounted for (Shahzaman et al., 2021; Tabassum et al., 2023). These unaddressed factors highlight the need for further research to develop a more nuanced understanding of drought impacts and vegetation dynamics in the UKDM. Moreover, the findings represent only a broad view of vegetation health across the district and lack the granularity needed for localised assessments. By incorporating precipitation data, additional environmental variables, and ground measurements, vegetation assessments could become more accurate, as integrating multiple drought indicators is essential for precise drought monitoring (Won & Kim, 2023) to inform local-level decisions.

## Results and discussion

**Fig. 3:**
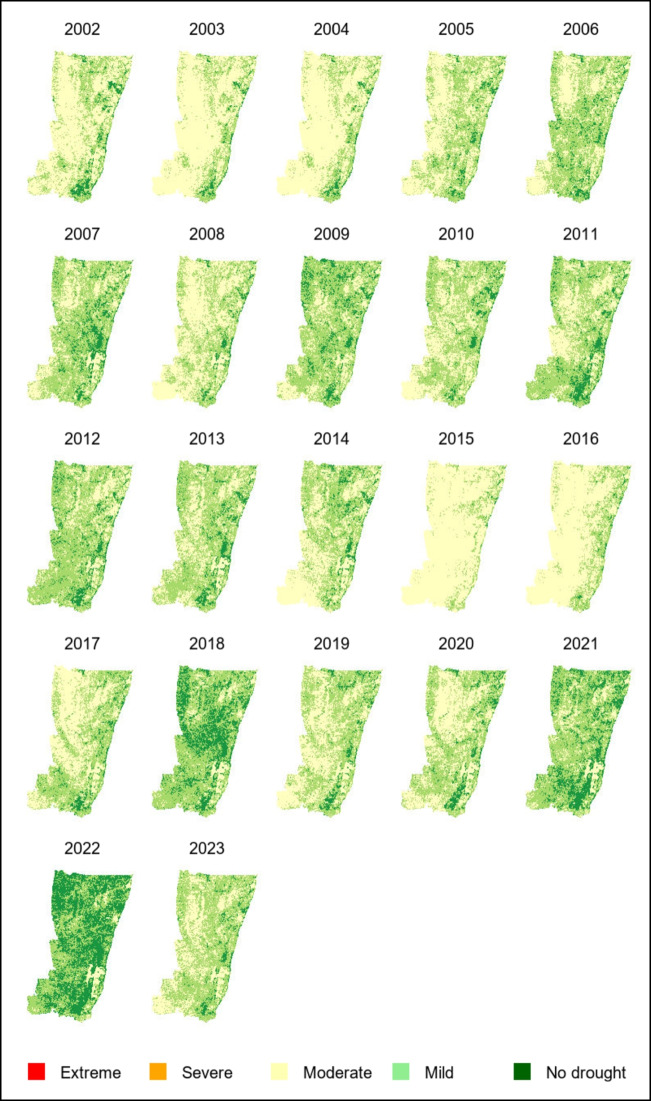
Spatial variation of the yearly (2002 to 2023) VHI for UKDM

Figure 3 depicts the spatial and temporal variation in VHI across the UKDM, highlighting trends and patterns that have emerged over two decades. A distinct east–west gradient is visible, with consistently higher VHI values in eastern areas indicative of healthier vegetation conditions. This pattern aligns with prior research, which attributes the robust vegetation health in these regions to the moisture-laden coastal climate influenced by the Indian Ocean (Sharma et al., 2022). Conversely, the inland western parts of the district display lower and more variable VHI values, suggesting higher drought vulnerability for agriculture as the moderating climatic influence of the ocean declines.

**Fig. 4:**
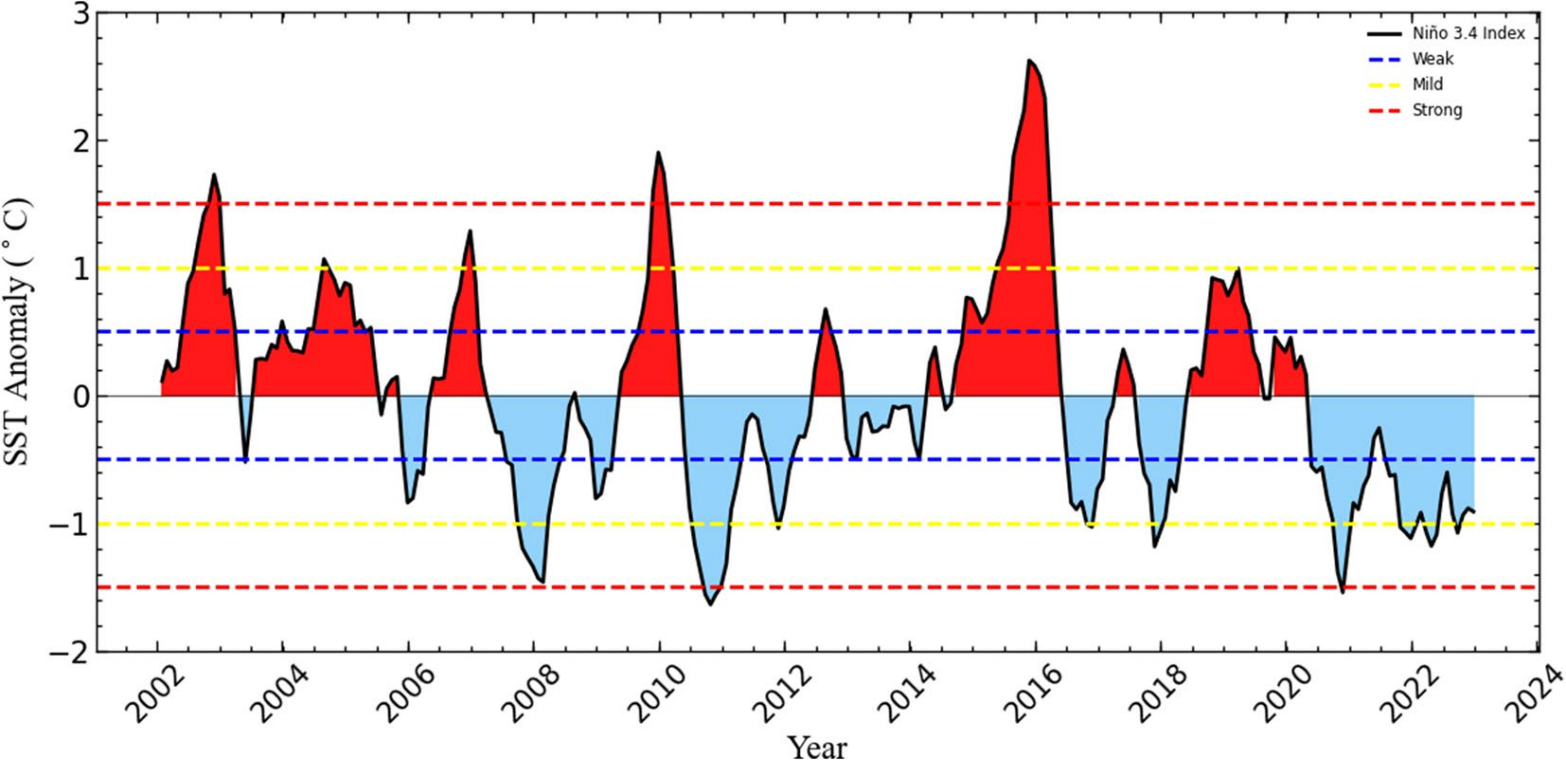
The standardised monthly Niño3.4 time series for the period from 2002 to 2023

Several years emerge as anomalously dry periods based on widespread depressed VHI. The years 2003, 2004, 2015, and 2016 show a substantially higher proportion of land surface experiencing moderate to severe agricultural drought compared to other years in the series. The VHI deterioration during 2015–2016 appears most extensive, coinciding with the most intense El Niño event across Southern Africa since 1997/1998 (Iwuji et al., 2023). This event and other significant El Niño years, 2003 and 2009/2010 (Fig. 4) underscore a pattern of increasingly frequent and intense droughts. These multi-year droughts highlight risks for farmers relying on rainfed agriculture across the UKDM. In contrast, the years including 2008, 2011, 2012, 2018, and 2020–2023 show predominantly green regions, indicating healthier vegetation and relatively favourable climatic conditions with adequate rainfall due to moderate and strong La Niña events.

*To identify the strength of the El Niño–Southern Oscillation (ENSO)events, the threshold is broken down into weak (0.5–0.9 SST anomaly), moderate (1.0–1.4 anomaly), and strong (≥ 1.5 anomaly) events. The El Niño events are shaded in red, whereas La Niña events are indicated in blue.*

**Fig. 5:**
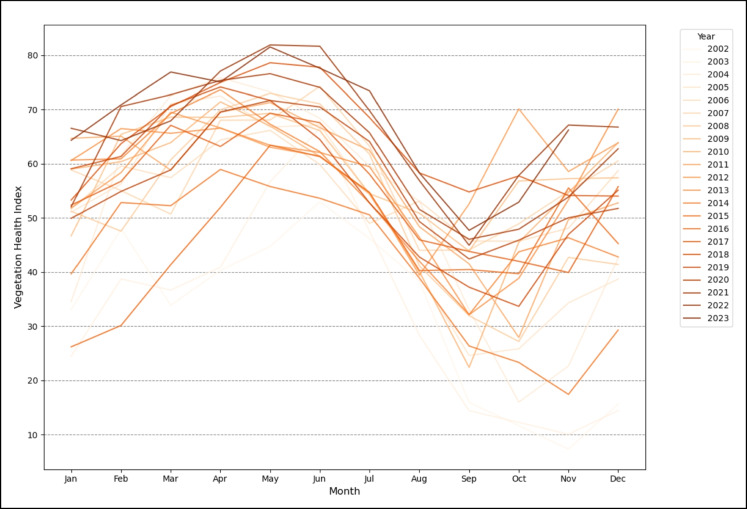
Monthly Mean VHI of UKDM from 2002 to 2023

The monthly averaged VHI values exhibit a distinct seasonal cycle, as illustrated in Fig. 5. The VHI is measured on a scale from 0 to 100, where values between 56 and 100 indicate robust vegetation health and values below 46 signal vegetation under stress. VHI values typically rise from an average of 50 in January to a peak between 70 and 80 from April to July. This upper range is often observed during periods with sufficient rainfall and moderate temperatures, promoting vigorous plant growth and high photosynthetic activity (Kogan et al., 2020). Following this peak, VHI values decline sharply around August to September and reache its lowest point between September and November, coinciding with the dry season when vegetation is under greater stress (Kogan et al., 2020). The subsequent increase in VHI values around December signal the onset of the next rainy period, demonstrating the responsiveness of vegetation to the resumption of rainfall. The fluctuations in vegetation health closely track the bimodal rainfall regime in South Africa, with vegetation stress rising during the winter and early spring dry spells (Dinku et al., 2018; Landman et al., 2012). In addition, Fig. 5 indicates considerable interannual variability in VHI values across the years, particularly evident during the dry season. While most years follow a similar seasonal pattern, some years (e.g. 2002, 2003, 2004, 2015, and 2016) exhibit lower VHI values, consistently below 40, especially from September to November. This suggests that these years experienced harsher drought conditions. In contrast, some years, such as 2022 and 2023, maintain VHI values above 50 throughout the year, reflecting more favourable climatic conditions and possibly higher-than-average rainfall, which supported healthier vegetation.

**Fig. 6:**
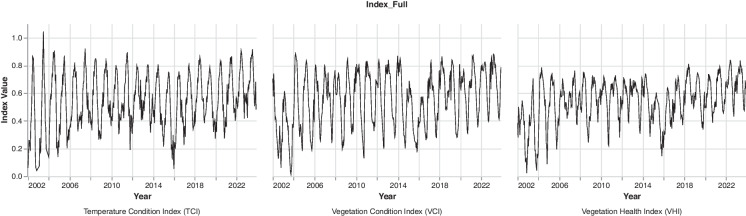
Yearly VCI, TCI, VHI for the years 2002 to 2023 for UKDM

The time series plots in Fig. 6 illustrate the fluctuation of vegetation health indices (VCI, TCI, and VHI) for the UKDM between 2002 and 2023. More broadly, the indices demonstrate a pattern of high interannual variability, with values oscillating between roughly 0.0 and 0.9 (on a scale from 0 to 1) during the study period. The consistent pattern of sharp declines in specific years (2003, 2015–2016) across all three indices reflects the occurrence of drought events. These drought episodes corroborate prior studies highlighting acute rainfall deficits and agricultural drought in KZN and eastern South Africa during this time. For example, Usman and Reason (2004) analysed meteorological data and found a peak in rainfall deficiency in October 2002, linked to El Niño conditions. Rouault and Richard (2005) reported a severe drop in water storage levels in major regional reservoirs in 2002–2003 tied to drought. The intense El Niño events in 2002–2004 as well as 2015–2016 triggered widespread drought across multiple regions, leading to devastating crop failures with severe humanitarian and agricultural impacts. The results of this study align with findings from previous research demonstrating that drought can severely impact agricultural production and food security. For example, earlier work by the Food Agricultural Organisation-FAO (2016) analysed the agricultural impacts of the 2015–2016 El Niño across Africa and found that maize yields fell by up to 50% in Southern Africa compared to the preceding seasons.

**Fig. 7:**
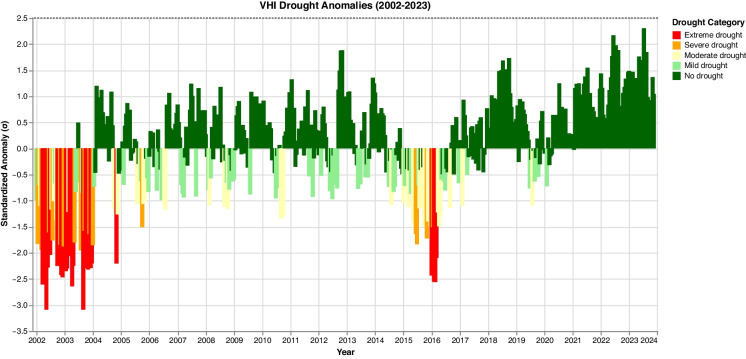
VHI anomalies classified by drought category between 2002 and 2023 for UKDM

Figure 7 illustrates fluctuations in VHI anomalies over two decades, with alternating periods of positive and negative anomalies. These anomalies provide insights into the temporal dynamics of drought and vegetation health variability and are crucial for identifying deviations from average vegetation conditions, helping to monitor drought onset, severity, and recovery. Positive anomalies indicate no drought, with vegetation health above the average conditions, while negative anomalies reflect varying levels of drought severity. Generally, positive VHI anomalies are more prevalent in most years, especially from 2006 onwards, with values often exceeding 0.5, suggesting a pattern where vegetation health remains in a ‘no drought’ state for much of the period. Conversely, the graph reveals two major drought episodes during 2002–2004 and 2015–2016, marked by extreme negative anomalies (below − 2.0), coinciding with strong El Niño events that caused significant rainfall deficits across Southern Africa. The findings resonate with Rouault and Richard (2005), who identified a peak drought period in KZN during 2004–2005. Another period of notable drought spanned 2015–2016, coinciding with the strong El Niño. In contrast, the last 3 years (2021–2023) show no appreciable VHI anomalies, indicating a recent reprieve from agricultural drought. However, cyclical drought patterns coupled with climate change projections of increased aridity in Southern Africa (Engelbrecht et al., 2015) suggest drought risks could escalate again in the coming years. Sustained monitoring of VHI anomalies is critical for drought early warning and preparedness in this region.

**Fig. 8:**
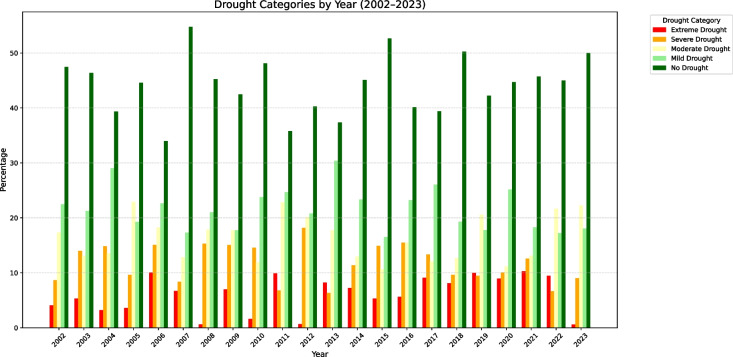
Distribution of drought categories per year (2002–2023) for UKDM

Further analysis of the full VHI time series (Fig. 8) shows that moderate and mild droughts are consistently prominent throughout the study period. This indicates underlying vulnerabilities that may be due to soil degradation, limited water availability, or localised climatic variability. Severe and extreme droughts are less frequent but still present in varying degrees across the years. The dominance of 'no drought' from 2006 to 2014 and again after 2020, with proportions exceeding 40-50% in these years suggests recovery periods likely supported by rainfall and cooler temperatures.

Studies by Usman and Reason (2004) link the early 2000s drought to the 2002–2003 El Niño event, which typically brings drier conditions to southern Africa. Chikoore and Jury (2021) have deconstructed South African drought periods and attributed them to climatic events, including the ENSO and the Indian Ocean Dipole (IOD), which disrupt typical weather patterns, leading to decreased rainfall. For example, the severe drought in 2016 aligned with the strongest recorded El Niño, causing a widespread reduction in precipitation across southern Africa. Positive phases of the IOD alter weather systems east of Madagascar, reducing moisture availability and contributing to drought conditions. Both ENSO and IOD usually peak during the southern hemisphere’s summer, intensifying their combined impact on the regional climate (Chikoore & Jury, 2021). In the UKDM specifically, Gwala (2018) documented extreme drought in 2002 and moderate to extreme drought in 2015, while Iwuji et al. (2023) identified 2014, 2015, and 2016 as dry years in Hlabisa under the Big 5 Hlabisa Local Municipality (LM), corroborating the findings in this study. These years coincide with major droughts across South Africa, including 2004–2005, 2008–2009, 2015–2016, and 2018–2020 (Meza et al., 2021). Moreover, the heightened severity of recent droughts in 2015, 2016, and 2019—surpassing those in 1983 and 1992—suggests a link to global warming (Chikoore & Jury, 2021). Rising temperatures increase evaporation rates, which dry out soils and diminish water resources. Ongoing monitoring is essential, as climate models suggest climate change may lead to more frequent and intense drought events in Southern Africa (Engelbrecht et al., 2015). Additionally, alterations in wind patterns—such as jet stream shifts, atmospheric subsidence, and anomalies in air currents—reduce atmospheric moisture. These changes hinder cloud formation and rainfall, further exacerbating drought conditions (Chikoore & Jury, 2021).

**Fig. 9:**
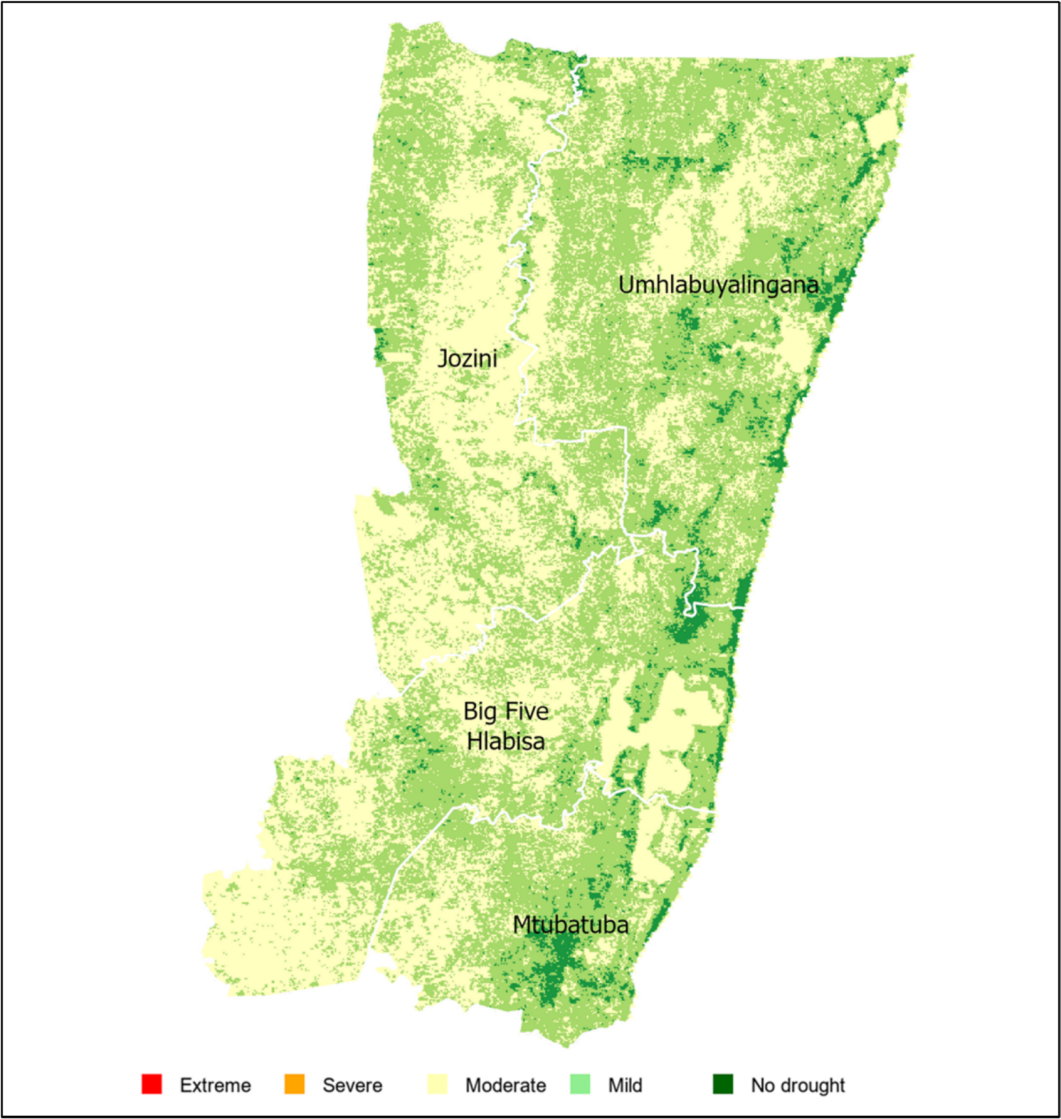
Spatial variation of the average VHI of UKDM for the period (2002–2023)

Figure 9 illustrates the spatial distribution of average VHI values across the UKDM over the study period. The map shows mild and moderate drought conditions dominate much of the landscape, particularly in inland areas such as Jozini and Big 5 Hlabisa LM, indicating widespread but minimal vegetation stress. These areas warrant targeted adaptation strategies. Localised coastal areas, particularly in Mtubatuba and parts of Umhlabuyalingana LM, exhibit ‘no drought’ conditions, likely due to higher rainfall and moderated temperatures influenced by the Indian Ocean.

Moderate drought conditions characterised most months across Mtubatuba, Umhlabuyalingana, Jozini, and Big 5 Hlabisa, ranging from 40.34% in Jozini to 59.75% in Umhlabuyalingana (Table 2). This indicates that vegetation health, as measured by VHI, frequently reflects moderate moisture stress in these areas. Mild drought conditions affect a smaller proportion of the area, with values ranging from 34.32% in Mtubatuba to 58.77% in Jozini, highlighting Jozini’s higher vulnerability to milder drought conditions. The presence of mild drought in all four LMs suggests that intermittent stress is common but less severe overall. ‘No drought’ conditions occur less frequently across the LMs, ranging from 0.89% in Jozini to 7.33% in Mtubatuba, indicating limited periods of optimal vegetation health in most areas. The lower percentage of ‘no drought’ conditions in Jozini suggests that this LM is more consistently affected by some level of drought compared to others.

**Table 2:** Overall mean percentage of drought categories in UKDM 2002–2023 as determined by VHI

Local municipality	Mild	Moderate	No drought
Mtubatuba	34.318	58.355	7.327
Umhlabuyalingana	34.897	59.749	5.354
Jozini	58.768	40.340	0.892
Big 5 Hlabisa	53.948	42.841	3.211

## Conclusion

Droughts are a recurring hazard in South Africa, presenting significant challenges to the agricultural sector and threatening food security and livelihoods. These events, while detrimental, also offer opportunities to improve drought risk mitigation and adaptation efforts as climate change intensifies (Meza et al., 2021). In this context, this study analysed the spatio-temporal drought variability in the UKDM using vegetation health indices (VCI, TCI, and VHI) to understand drought impacts better and inform adaptation strategies. These indices offer valuable insights into vegetation stress and health, which are directly influenced by drought conditions. The analysis revealed distinct seasonal fluctuations in the VHI and identified significant historical droughts, such as those occurring between 2002–2004 and 2015–2016. These episodes were largely associated with El Niño events, known to affect precipitation patterns in Southern Africa and exacerbate drought conditions. Through spatial analysis, the study pinpointed specific areas within the district with heightened susceptibility to drought, including Jozini and Big 5 Hlabisa LM. The effectiveness of VHI in capturing known drought events underscores its potential as a reliable monitoring tool for drought assessment.

Small-scale agriculture, which supports a large proportion of the rural population, is particularly vulnerable. The impacts of drought exacerbate food insecurity, malnutrition, and poverty and therefore hinder efforts to achieve sustainable development goals (Meza et al., 2021). Based on the study findings, it is recommended that vegetation health indices be used for real-time drought monitoring to improve the accuracy and timeliness of early warning systems. Spatially explicit drought data should be employed to identify and prioritise vulnerable areas, with targeted interventions focused on chronic hotspots such as inland areas of Jozini and Big 5 Hlabisa LM. Proactive adaptation strategies may include the introduction of drought-resistant crop varieties, investing in irrigation infrastructure, and implementing integrated water resource management practices. These measures will enhance the resilience of the agricultural sector, safeguard rural livelihoods, and contribute to long-term food security amidst the growing challenges posed by climate change.

## Data Availability

The author confirms that all data generated or analysed during the study can be requested from the corresponding author.
